# *Notes from the Field:* Rebound in Routine Childhood Vaccine Administration Following Decline During the COVID-19 Pandemic — New York City, March 1–June 27, 2020

**DOI:** 10.15585/mmwr.mm6930a3

**Published:** 2020-07-31

**Authors:** Marisa Langdon-Embry, Vikki Papadouka, Iris Cheng, Mohammed Almashhadani, Alexandra Ternier, Jane R. Zucker

**Affiliations:** ^1^Bureau of Immunization, New York City Department of Health and Mental Hygiene; ^2^Immunization Services Division, National Center for Immunization and Respiratory Diseases, CDC.

Concerns have been raised about falling childhood vaccine administration and vaccination coverage rates (*1*,*2*) during the coronavirus disease 2019 (COVID-19) pandemic. In New York City (NYC), decreasing vaccination coverage has been of particular concern in light of recent outbreaks of vaccine-preventable diseases, including a large measles outbreak during 2018–2019 (*3*). The effect of the COVID-19 pandemic on routine childhood vaccination was monitored by the NYC Department of Health and Mental Hygiene (DOHMH) using the Citywide Immunization Registry (CIR),* a population-based immunization information system with high data quality and provider participation (*4*,*5*). CIR includes 2.7 million patient records for NYC persons aged 0–18 years and receives reports from approximately 1,600 immunization facilities. The weekly number of routine childhood vaccine doses administered to persons aged <24 months and 2–18 years in 2020 was compared with the number administered during the same period in 2019; influenza vaccine and vaccines administered in pharmacies and hospital nurseries were excluded from this report.^†^ Likewise, the weekly number of unique facilities that reported administering at least one childhood vaccine in 2020 to 2019 was also compared.

A decrease in the number of vaccine doses administered in NYC was detected beginning the week of March 8, 2020, 1 week after the first COVID-19 case was confirmed in NYC. Those numbers declined further after the New York State on PAUSE Executive Order^§^ went into effect on March 22, which required New Yorkers to stay at home to reduce the spread of SARS-CoV-2, the virus that causes COVID-19. The largest relative decrease was observed during the week of April 5–11 and was less pronounced in persons aged <24 months (62% decrease, from 33,261 doses in 2019 to 12,746 doses in 2020) than in those aged 2–18 years (96% decrease, from 23,631 doses in 2019 to 1,054 doses in 2020) (Figure). During that same week, 488 facilities reported administering at least one vaccine to a person aged <24 months, representing a 46% decrease from the 900 reporting immunization data during the same period in 2019; the number of facilities that reported administering at least one vaccine to a person aged 2–18 years decreased 78%, from 1,238 in 2019 to 275 in 2020.

**FIGURE:**
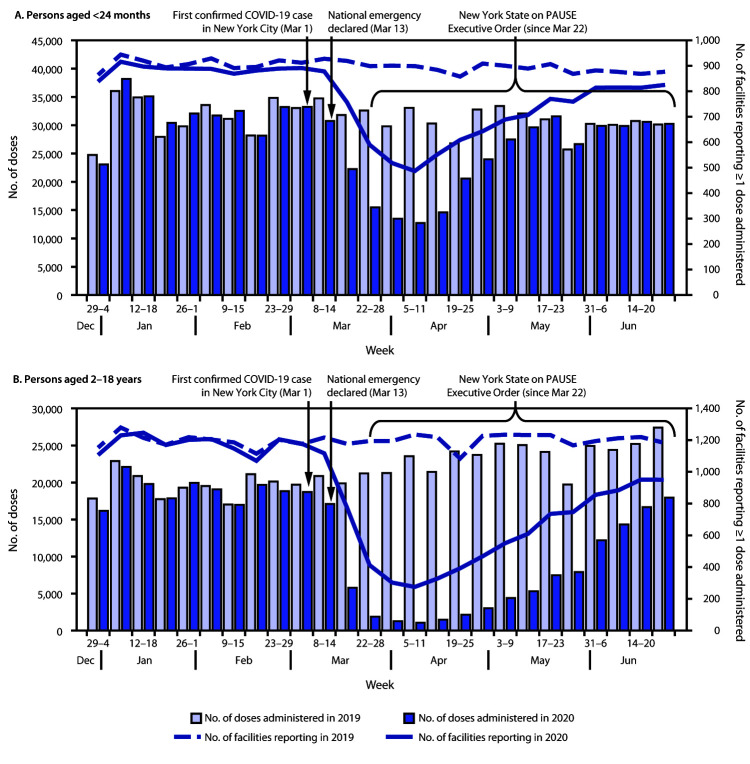
Routine childhood vaccine doses administered, by week*^,†^ — New York City, December 2019–June 2020^§^ **Source:** New York City Department of Health and Mental Hygiene Citywide Immunization Registry (CIR); data are as of July 14, 2020. **Abbreviation:** COVID-19 = coronavirus disease 2019. * Vaccine doses administered during December 29, 2019–June 27, 2020, and entered into CIR by July 12, 2020, compared with vaccine doses administered during December 30, 2018–June 29, 2019, and entered by July 14, 2019. Week format (Sunday–Saturday) is based on dates in 2020. ^†^ Excludes influenza vaccine and immunizations administered in pharmacies and hospital nurseries. ^§^ The New York State on PAUSE Executive Order went into effect at 8:00 p.m. on Sunday, March 22, 2020, and required New Yorkers to stay at home to reduce the spread of SARS-CoV-2. https://www.state.gov/wp-content/uploads/2020/03/2020-03-20-Notice-New-York-on-Pause-Order.pdf.

In response to the decline in vaccine administration documented during the COVID-19 pandemic, the NYC DOHMH sent three letters and one Health Alert Network notification to health care providers during March–June highlighting the importance of continuing routine immunization. In May, messages were placed on the CIR’s vaccine ordering module to encourage providers to order sufficient vaccine to catch up their unvaccinated patients. Reminder and recall tools available in the CIR’s provider portal were promoted to identify and recall children who were overdue for vaccination. The importance of childhood vaccination was the subject of a mayoral press conference on May 20 that was widely covered by local media.^¶^ A webinar targeting NYC pediatric health care providers was held on June 17 to promote strategies to increase vaccination.

Vaccine administration increased among persons aged <24 months starting the week of April 19–25, as the number of new COVID-19 cases declined,** and returned to levels comparable with those during 2019 beginning the week of May 17 (Figure). During the most recent week for which data were available (June 21–27), the number of facilities that reported administering at least one vaccine to a person aged <24 months increased 69% from the lowest point to 825. Vaccine administration among persons aged 2–18 years increased starting the week of April 26–May 2 and has continued to rise, but as of June 27 still had not reached levels comparable with 2019 (Figure). During the week of June 21–27, 35% fewer vaccines were administered to persons aged 2–18 years than were administered during the same week in 2019 (17,947 doses versus 27,405). The number of facilities that reported administering at least one vaccine to a person aged 2–18 years increased to 950, approximately three times as many as at the lowest point during 2020 (275 facilities).

The increase in vaccine administration seen in May and June is encouraging, and DOHMH continues to promote routine childhood vaccination using methods including public service announcements and letters, guidance, and webinars for health care providers on strategies to encourage parents to catch up their children’s vaccinations. The rebound of administration of routine early childhood vaccines in NYC demonstrates the critical role of public health departments and partnerships with numerous stakeholders, specifically the provider community, in childhood vaccination. The availability of an immunization infrastructure to rapidly communicate with providers, an effective immunization information system to identify unvaccinated children, and the Vaccines for Children Program^††^ provider and vaccine distribution network have all been important to NYC’s response and will be critical to distribution and administration of COVID-19 vaccines when they become available.

## Footnotes

* CIR is a database of immunization records and birth records. All vaccine doses administered to persons aged ≤18 years in NYC are required to be reported to CIR within 14 days of administration according to the NYC Health Code and New York State Public Health Law. Birth certificates are uploaded to CIR twice per week.

† Pharmacies and hospital nurseries were excluded because they do not administer routine childhood immunizations in NYC. In New York State, pharmacies are only authorized to administer influenza vaccine to persons aged 2–18 years. Hospital nurseries only administer the hepatitis B birth dose to infants.

§ https://www.governor.ny.gov/news/governor-cuomo-issues-guidance-essential-services-under-new-york-state-pause-executive-order.

¶ https://www.ny1.com/nyc/all-boroughs/news/2020/05/20/mayor-urges-parents-to-vaccinate-kids-during-pandemic-.

** https://www1.nyc.gov/site/doh/covid/covid-19-data.page.

†† https://www.cdc.gov/vaccines/programs/vfc/index.html.
